# Transcriptomic Analysis of MDBK Cells Infected with Cytopathic and Non-Cytopathic Strains of Bovine Viral Diarrhea Virus (BVDV)

**DOI:** 10.3390/v14061276

**Published:** 2022-06-11

**Authors:** Paweł Mirosław, Marzena Rola-Łuszczak, Jacek Kuźmak, Mirosław P. Polak

**Affiliations:** 1 Department of Virology, National Veterinary Research Institute, 24-100 Pulawy, Poland; ppolak@piwet.pulawy.pl; 2 Department of Biochemistry, National Veterinary Research Institute, 24-100 Pulawy, Poland; jkuzmak@piwet.pulawy.pl; 3 Department of Omics Analysis, National Veterinary Research Institute, 24-100 Puławy, Poland

**Keywords:** bovine viral diarrhea virus, microarrays, transcriptome, cell culture

## Abstract

Bovine viral diarrhea virus (BVDV) belongs to the *Flaviviridae* family and the *Pestivirus* genus. Infection with BVDV causes a disease with a wide spectrum of clinical symptoms, most often mild, although infections with this virus constitute a serious economic problem all over the world. The virus is characterized by a high genetic variability, while the accumulation of single mutations leads to the formation of its new variants. The aim of this study was to better understand the complicated pathogenesis of this disease at the molecular level via the analysis of the transcriptome of cells infected with this virus. The bovine kidney cell line (MDBK), the cytopathic (cp) reference strain, and two non-cytopathic (ncp) BVD virus field strains were used in transcriptomic studies. The cell transcriptome was tested 24 and 72 h after infection. The results of the microarray analysis revealed changes in the expression levels of numerous genes. Genes with changed expression as a result of infection with the cp strain caused changes in the expression levels of a large number of genes and enriched a number of pathways. Genes with increased expression levels were enriched among other pathways involved in the cell cycle, while genes with reduced expression levels enriched pathways mostly related to metabolism. Genes with increased expression levels as a result of infection with ncp strains enriched a much smaller number of pathways, among them, pathways related to signaling activity 24 h post-infection and serine biosynthetic pathways both 24 and 72 h post-infection. Pathways enriched by genes with reduced expression levels were related to the innate immune response (72 h post-infection) or metabolism (24 and 72 h post-infection). The results of microarray studies can help us to better understand the host’s response to BVDV infection.

## 1. Introduction

The bovine viral diarrhea virus (BVDV) is one of the most common pathogens in cattle farming around the world, causing significant economic losses [1]. Bovine viral diarrhea virus is a member of the *Pestivirus* genus in the *Flaviviridae* family. Two different BVDV species have been recognized: BVDV-1 (pestivirus A) and BVDV-2 (pestivirus B) [2].

BVDV can cause a variety of clinical signs in infected animals. An acute infection is usually mild and can go unnoticed. The main symptoms are transient leukopenia, mild fever, diarrhea, increased nasal discharge, cough, and other mild respiratory symptoms. In pregnant cows, abortions may occur [3]. An important issue is immunosuppression caused by the BVD virus, which significantly increases the risk of superinfection with other pathogens [4]. Infection between 40 and 120 days of pregnancy can lead to the birth of a persistently infected (PI) calf. PI animals are the main source of the virus in the herd. Moreover, they often do not show any clinical signs of infection. The transmission of BVDV to another individual can occur through direct contact between animals, or indirectly through contact with infectious secretions, contaminated feed, or by an iatrogenic route, among other possibilities. Pestiviruses are present in all excretions and secretions of persistently infected animals (e.g., saliva, tears, nasal secretions, blood, milk, urine, feces, semen) [3].

The high variability of the virus resulting from the imprecise replication of the viral genome and the accumulation of single mutations leads to the formation of subsequent virus subtypes. The same process makes the design of fully effective vaccines difficult [5].

Two biotypes of BVDV are recognized: cytopathic (cp) and non-cytopathic (ncp). cp strains induce a cytopathic effect in infected cell culture, while ncp strains multiply without visible changes. There is a link between RNA recombination and the appearance of cp viruses [6]. The infection of a PI individual by a cp strain can lead to the development of a deadly mucosal disease (MD). BVDV can employ two survival strategies, including the ‘infect-and-persist’ strategy employed by ncp strains and the ‘hit-and-run’ strategy employed by cp or highly virulent ncp strains [7]. The first strategy allows ncp strains to successfully evade the host’s immune system through a variety of mechanisms. 

Innate immunity plays an important role in the host’s response to infectious agents as the first line of defense. The regulation of many processes is activated by pathogen-associated molecular patterns (PAMPs), i.e., specific structural motifs of molecules associated with a given pathogen. The binding of appropriate viral structures to the PRRs (pattern recognition receptors) causes the activation or inhibition of signaling pathways by inducing an appropriate pattern of gene expression. The induction of innate antiviral immunity severely restricts viral replication, and viruses have developed various strategies to avoid this. The N^pro^ pestivirus protein prevents the formation of interferon-α/β in infected cells via proteasomal degradation of interferon regulatory factor 3 (IRF3), which is an interferon-β transcription factor [8]. It has also been shown that interferon regulatory factor 7 in plasmocytic dendritic cells is blocked by the N^pro^ protein [9]. The interaction of N^pro^ and antiapoptotic protein HAX-1 was confirmed using coprecipitation assays [10]. E^rns^ can bind to exogenous dsRNA and inhibit dsRNA-induced production of IFN-β upstream in the TLR3-initiated signaling pathway [11]. The interaction of NS5A with NIBP (bovine NIK- and IKKbeta-binding protein), which is involved in protein trafficking and nuclear factor kappa B (NF-κB) signaling in cells, was also confirmed [12].

The mechanisms underlying the pathogenesis and host interaction with BVDV are complex. While several strategies for avoiding the immune system response by BVD virus are involved, especially the innate immune system, the essence of the full mechanisms underlying BVDV–host interactions during infection is still not fully understood.

Recently, microarray technologies have provided the opportunity to produce large amounts of data. This has allowed for thorough analyses of host defense mechanisms and immunity avoidance strategies at the whole mRNA level in the event of infections with both cp and ncp strains.

The aim of this study was to investigate changes in gene expression levels and to identify activated or suppressed metabolic and signaling pathways at 24 and 72 h post-infection of cell culture with BVD virus.

## 2. Materials and Methods

### 2.1. Viruses and Cells

Madin–Darby bovine kidney (MDBK) cells from the American Type Culture Collection (ATCC) were cultured in Minimal Essential Medium Eagle (MEM-E) (Sigma-Aldrich, Saint Louis, MI, USA) supplemented with 10% fetal bovine serum (FBS) (Gibco, Billings, MT, USA) (free from antibodies, BVD virus, and mycoplasma *contamination)* and 1% antibiotics (Sigma-Aldrich, Saint Louis, MI, USA). MDBK cells were maintained at 37 °C in an atmosphere of 5% CO_2_.

MDBK cells were plated onto T25 cell culture flasks (Thermo Scientific, Rochester, NY, USA). After 24 h, MDBK cells were inoculated with BVDV with MOI = 0.05 and maintained at 37 °C with 5% CO_2_. Two non-cytopathic BVD virus field strains were used for experimental infection, one belonging to subtype 1f (strain 59-GB/11) and the other belonging to subtype 1b (strain 60-GB/11), as well as the cytopathic reference strain NADL (subtype 1a) from ATCC. Two T25 bottles with cell culture were infected with each strain. RNA from the first flask was isolated 24 h after inoculation and from the second one after 72 h. In parallel, 2 flasks contained uninfected cell culture. Then, a biological repetition of the experiment was performed.

### 2.2. Microarray Analysis

Total RNA was extracted from the cells 24 or 72 h post-infection using a Qiagen RNeasy Mini Kit (Qiagen, Hilden, Germany) according to the manufacturer’s instructions. RNA concentration and RNA integrity number (RIN) measurements were carried out using the 2100 Bioanalyzer and Agilent RNA 6000 Nano Kit (Agilent Technologies, Santa Clara, CA, USA). Samples with RIN values ranging from 8 to 10 were used for further analysis.

Briefly, 50 ng of total RNA from each sample was reverse-transcribed to generate cDNA and then transcribed into Cy3-labeled cRNA (samples obtained from uninfected cells) and into Cy5-labeled cRNA (samples obtained from infected cells) with a Low-Input Quick Amp Labeling Kit (Agilent Technologies, Santa Clara, CA, USA). An RNA Spike-In Kit was used as an internal control. After purification of labeled RNA (Qiagen RNeasy Mini Kit, Qiagen, Hilden, Germany), the yield (ng of cRNA) and specific activity (pmol of Cy3 or Cy5/µg of cRNA) were quantified using NanoDrop One (Thermo Scientific, Wilmington, DE, USA). Here, cRNA fragmentation was performed using the Gene Expression Hybridization Kit (Agilent Technologies, Santa Clara, CA, USA). The composition of the reaction mixture was as follows: 825 ng Cy3-labeled cRNA, 825 ng Cy5-labeled cRNA, 11 μL 10× Blocking Agent, and 2.2 μL of 25× Fragmentation Buffer. Nuclease-free water was added to the mixture to obtain a total volume of 55 µL. The samples were incubated for 30 min at 60 °C, then 55 µL of GEx HI-RPM buffer was added to stop fragmentation. Two technical repeats of the experimental setup were performed, which together with the biological repeats allowed for the preparation of 24 arrays. Then, samples were hybridized on Agilent arrays (Bovine (v2) Gene Expression 4 × 44K Microarray) for 17 h at 65 °C in Agilent hybridization chambers rotating at 10 rpm.

After hybridization, the arrays were subsequently washed twice with GE wash buffer 1 for 1 min at room temperature and GE wash buffer 2 for 1 min at approximately 37 °C. After washing, slides were scanned using a SureScan D× Microarray Scanner (Agilent Technologies, Santa Clara, CA, USA). Images obtained after scanning were analyzed using Agilent Feature Extraction software (version 12.0.3.1) (Agilent Technologies, Santa Clara, CA, USA. A detailed analysis was performed including filtering of outlier spots, background subtraction, and dye normalization (linear LOWESS). The raw and processed data were deposited in NCBI’s Gene Expression Omnibus (GEO) with accession numbers GSE166288 for cp and GSE166528 for ncp strains. The microarray experiment was performed according to the MIAME standards (minimum information about a microarray experiment) [13].

### 2.3. Statistical Analyses

The statistical analysis was performed using Gene Spring 14.8 software (Agilent Technologies, Santa Clara, CA, USA). The statistical significance of the differences was evaluated using Student’s *t*-test. A moderated *t*-test was used to compare two groups of BVDV-1b-infected cell versus BVDV-1f-infected cells. Multiple testing correction was performed using Benjamini and Hochberg False Discovery Rate (FDR) tests. Entities with FDR ≤ 0.05 and fold changes expressed as |FC| ≥ 1.5 were considered significant. The entities for which the official gene symbols were assigned and recognized as DEGs (differentially expressed genes) were subjected to enrichment analyses of Gene Ontology (GO) and KEGG pathways using DAVID 8.6 [14]. Fisher’s exact test was used for the enrichment analyses. GO terms and pathways with *p*-values ≤ 0.05 were considered significantly enriched.

### 2.4. Differential Expression Analysis

A PCA (principal component analysis) and hierarchical clustering analysis were used as a quality control step to indicate the similarities and differences between the analyzed samples and biological groups (GeneSpring 14.8) (Agilent Technologies, Santa Clara, CA, USA). On a PCA plot, samples with similar expression profiles were positioned in proximity to each other.

In order to answer the question of whether the classification of samples based on a list of entities coincides with the theoretical classification, the main components analysis, namely the PCA, was used, which indicates the similarities and differences between the analyzed samples resulting from the components being the basic sources of variance in the experiment.

**Figure 1:**
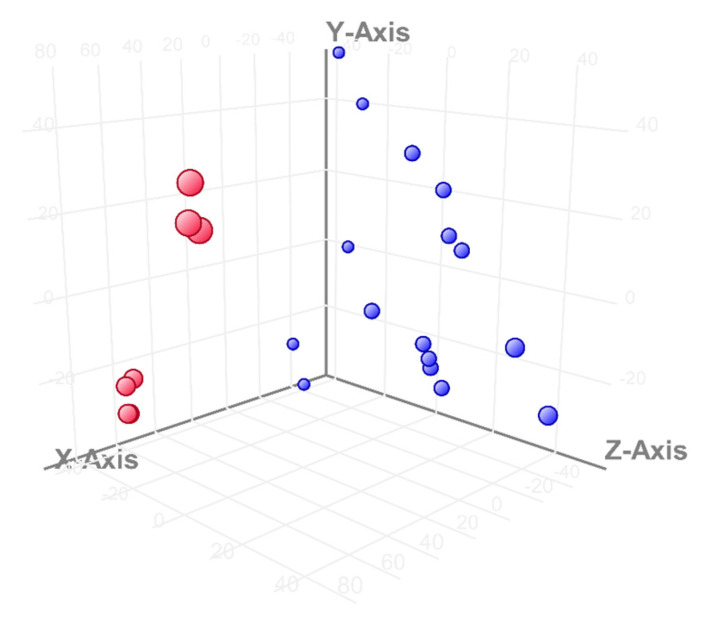
The differences in expression profiles investigated via PCA. The expression profiles caused by infection with the cp strain are shown in red and with ncp in blue.

**Figure 2:**
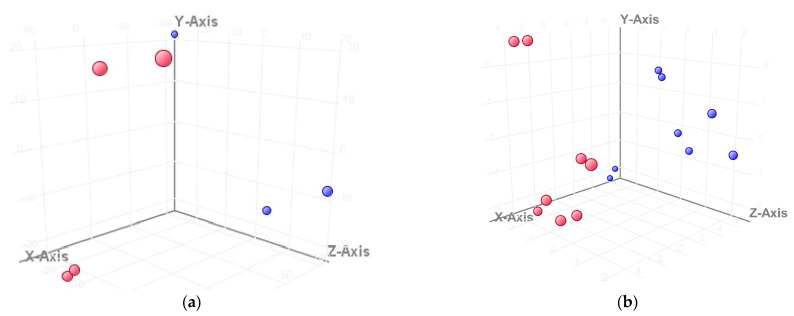
The differences in expression profiles investigated by PCA. The samples from the first day after infection with cp (**a**) and ncp (**b**) strains are marked in red, while the ones from the third day after infection are marked in blue. The samples from the first day post-infection are gathered together in a different area than the samples from the third day post-infection.

Figure 1 shows the principal component analysis, whereby the differences between the lists of entities after infection with cp and ncp strains are clearly visible. In Figure 2, we can see that the expression profiles cluster in different areas of the plot, depending on which day after infection they come from.

One array representing the transcriptional profile of cells infected with the cp strain 72 h p.i. was unsealed during hybridization. The PCA showed that the data from this array differed significantly from the other arrays (Figure S1); therefore, to analyze the changes in the gene expression of cells infected with the cp strain after 72 h of incubation, 3 arrays instead of the planned 4 were used.

Venn diagrams were used to show differences between the numbers of significant DEGs of cells infected with different biotypes and between different periods after infection, as well as the overlap between each set of genes [15].

### 2.5. Quantitative Real-Time RT-PCR (RT-qPCR)

Reverse-transcription quantitative real-time PCR (RT-qPCR) was performed to confirm the changes in expression of several genes from microarray analyses of all tested groups. Three genes (CXCL8 (IL-8), PSPH, GALNT18) that were found to be differentially expressed in some experimental systems were selected. The relative level of change in their expression was tested in each experimental system. Results were normalized using beta-2-microglobulin (B2M) as a reference gene. The list of primers used in the study is shown in Table 1. Total RNA samples derived from cell culture samples were used, as in the microarray study, representing both times after infection as well as each experimental setup, i.e., cp strain and ncp strain inoculations plus uninfected cells.

For analysis of the correlations between fold changes obtained from microarray and those from RT-qPCR, Spearman r correlation coefficients and *p*-values were determined to replicate and build on the published results.

Here, 1 µg samples of RNA from infected and uninfected cell culture were digested with DNase I Amplification Grade (Invitrogen, Carlsbad, CA, USA) and reverse-transcribed using an NG dART RT kit (EURX, Gdansk, Poland) according to the manufacturer’s instructions. The synthesis of cDNA was performed at 50 °C for 60 min. Two types of reverse-transcription reactions were performed for each sample: one with the RT enzyme, for which an appropriate material was obtained for expression testing, and the other without the enzyme to confirm the absence of residual DNA. Real-time PCR (qPCR) was performed on a ROCHE LightCycler 96 instrument (Roche, Mannheim, Germany). The QuantiTect SYBR Green PCR Kit (Qiagen, Hilden, Germany ) was used, and the mixture was as follows: 10 µL 2× Quantitect Mix, 1 µL of primer at 10 µM, 6 µL of water, and 2 µL of cDNA. Every qPCR reaction was performed in duplicate (technical replicates) using a thermal profile: 95 °C for initial denaturation and 45 cycles of 15 s denaturation at 94 °C, 30 s of primer annealing at 60 °C, and 30 s extension at 72 °C. The efficiency of each reaction was determined based on a serial dilution of pooled cDNA templates (1:10, 1:100, and 1:1000) within the expected range at 90–110% efficiency. Relative gene expression levels were calculated using the method described by Pfaffl [19].

For analysis of correlations between fold changes obtained from microarray and RT-qPCR analyses, Spearman r correlation coefficients and *p*-values were determined.

## 3. Results

### 3.1. Differential Expression Analysis

**Figure 3:**
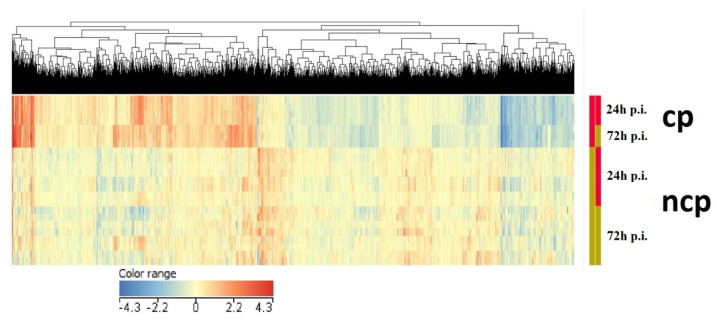
Differences in expression represented by a heat map created via hierarchical cluster analysis using a list of entities with statistically significantly altered expression levels (FDR ≤ 0.05). The expression profiles induced by infection with the cp strain are marked in red with the ncp strains marked in gold in the first column. In the second column, the red rectangle shows the gene expression profiles induced at 24 h p.i., while the gold rectangle shows expression profiles induced at 72 h p.i. Reduced expression values are marked in blue and the degrees of reduction are presented with individual shades, while increased expression is shown in red with corresponding shades.

Figure 3 shows a hierarchical cluster analysis of the differentially expressed list of entities. The figure clearly shows the differences in the expression profiles between the cp strain and the ncp strains. The clusters of entities distinguished by this approach included those with changed expression profiles after BVDV infection.

A moderated Student’s *t*-test (FDR ≤ 0.05) showed that the differences in the levels of gene expression caused by the BVDV-1b and BVDV-1f strains were minimal. It was possible to identify only four entities, including CXADR, MFSD11, and SLC44A1, which were characterized by statistically significantly higher expression levels in cells infected with the BVDV-1b strain as compared to BVDV-1f (Table S1). Because of the small changes in the cell culture caused by viruses of the ncp biotype and functional differences caused by individual subtypes, we decided to combine the 1f and 1b subtypes into one group, ncp strains, increasing the number of samples and the statistical power of the test.

Here, 24 h after infection with the NADL strain, 5200 entities were identified with changed expression levels against the control. Among these, 2369 were overexpressed, while a decrease in expression was noted for 2831 entities. Next, 72 h after infection, compared to the control, changes were observed for 4375 entities. Among these, 2180 were up- and 2195 were downregulated. For the complete lists of significantly differentially expressed entities after infection with the cp strain, see Tables S2 (24 h p.i.) and S3 (72 h p.i.). Official gene symbols were assigned to 3505 entities 24 h p.i., whereby 1683 genes were upregulated and 1822 were downregulated. After 72 h p.i., for 1592 genes, an increase in expression was noticed and for 1405 genes, a decrease in expression was noticed. The most noticeable changes were the genes encoding the chemokines GRO1 (CXCL1) (7.2; 7.59) and CXCL8 (IL-8) (6.31; 6.93). These showed the greatest increases in expression levels both 24 and 72 h after infection (the log2FC values in brackets are presented for 24 and 72 h post-infection). The greatest decrease in expression 24 h after infection was observed for the gene encoding asporin (ASPN) (−4.52). The gene whose expression was most reduced 72 h after infection was the gene encoding glutathione S-transferase A2 (GSTA2) (−5.58). 

Infection with ncp field strains 24 h post-infection resulted in differential regulation of 1125 entities. There was an increase in expression for 797 entities and a decrease for 328. After 72 h, a difference in expression was observed for 980 entities. Among them, the expression of 617 increased and the expression of 363 decreased. For the complete lists of significantly differentially expressed entities after infection with the ncp strains, see Tables S4 (24 h p.i.) and S5 (72 h p.i.). Official gene symbols were assigned to 646 entities 24 h p.i. (469 increased expression, 177 decreased). At 72 h p.i., 408 genes were increased in expression, while 234 were decreased. The genes with the most increased expression 24 h after infection were serum amyloid A3 (SAA3) (1.95) and the gene encoding glycine decarboxylase (GLDC) (1.83); the gene encoding the scavenger receptor A5 (SCARA5) (−1.35) was characterized as having the most decreased expression 24 h after infection. Furthermore, 72 h after infection, the expression levels of the metallothioneins metallothionein 2A (MT2A) (3.03) and metallothionein 1A (MT1A) (2.58) increased the most, while the lowest FC index was found for the gene encoding the calcium-binding protein S100G (−2.64). Higher changes in gene expression (higher absolute FC values) were seen 72 h after infection in the case of infection with both the cp strain and the ncp strains.

**Figure 4:**
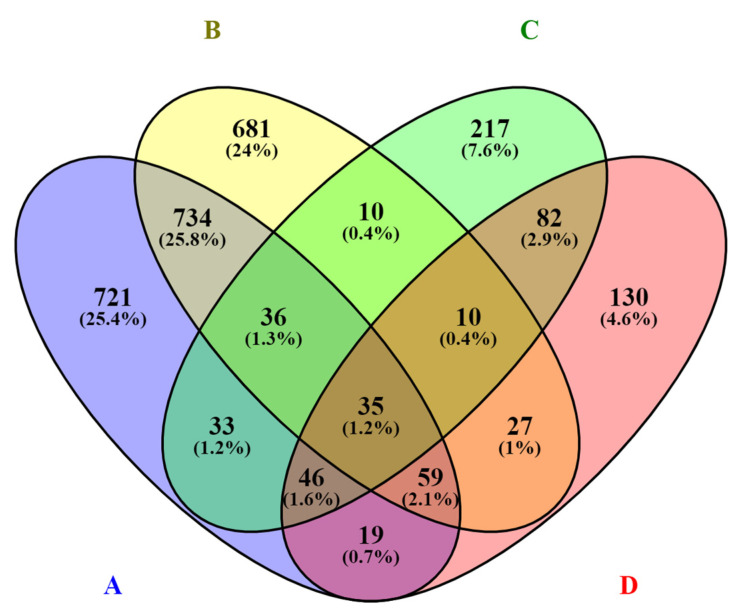
Venn diagram showing the number (FC ≤ 1.5; FDR ≥ 0.05) and relationship between transcripts with increased expression due to infection with individual BVDV biotypes. Number of transcripts induced by: A—biotype cp 24 h p.i., B—biotype cp 72 h p.i., C—biotype ncp 24 h p.i., D—biotype ncp 72 h p.i.

**Figure 5:**
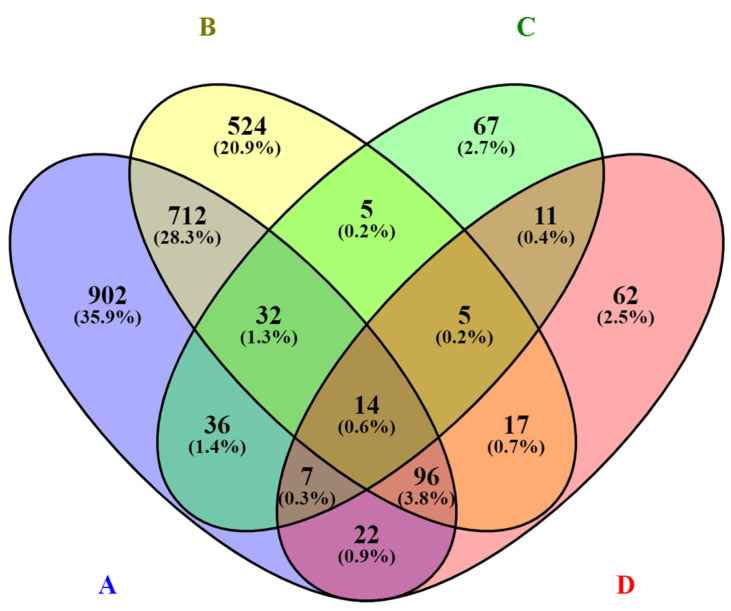
Venn diagram showing the number (FC ≤ 1.5; FDR ≥ 0.05) and the relationship between transcripts with decreased expression due to infection with individual BVDV biotypes. Number of transcripts induced by: A—biotype cp 24 h p.i., B—biotype cp 72 h p.i., C—biotype ncp 24 h p.i., D—biotype ncp 72 h p.i.

Venn diagrams show correlations between groups. Here, 75.2% of all genes whose expression levels increased were related only to the infection with the cp strain, 15.1% of transcripts were assigned exclusively to cells infected by ncp strains, and 9.9% of transcripts were common for both groups (Figure 4). Similarly, for genes whose expression levels decreased, 85.1% of all genes were associated with infection with the cp strain. Only 5.6% of the transcripts were infection-specific for ncp strains; 9.4% of the transcripts were common to both groups (Figure 5).

After the infection with the cp strain, more genes changed expression levels in comparison to those infected with the ncp strains, confirming the much greater influence on the functioning of the cells than the infection with the ncp strains.

Both cp and ncp strains induced changes in the expression of more genes 24 h after infection than 72 h p.i. However, in the case of ncp strains, these changes were minor. In both cases, a greater number of genes decreased their expression 72 h p.i. as compared to 24 h p.i.

The gene ontology analysis is presented below. Annotations were enriched with DEGs after infection, and three from each group (BP, MF, CC) with the lowest *p*-values are listed in the text and Table 2. The upregulated DEGs obtained 24 h after infection with the cp strain were annotated with 263 GO terms including cell division (BP), mitotic nuclear division (BP), cell cycle (BP), cytoplasm (CC), nucleus (CC), spindle pole (CC), ATP binding (MF), DNA binding (MF), and histone binding (MF), and 72 h post-infection with cp strain 166 terms were annotated with cell division (BP), regulation of cell proliferation (BP), apoptotic process (BP), cytoplasm (CC), nucleus (CC), spindle pole (CC), microtubule binding (MF), ATP binding (MF), and protein kinase binding (MF). The downregulated DEGs 24 h p.i. with cp strain were annotated with 145 GO terms, including sphingolipid biosynthetic process (BP), antigen processing and presentation of peptide or polysaccharide antigen via MHC class II (BP), protein glycosylation (BP), lysosomal membrane (CC), extracellular exosome (CC), lysosome (CC), monooxygenase activity (MF), heme binding (MF), and serine-type carboxypeptidase activity (MF). Furthermore, 72 h post-infection with cp strain (118 terms), the genes were annotated with metabolic process (BP), ATP-synthesis-coupled electron transport (BP), response to oxidative stress (BP), extracellular exosome (CC), lysosome (CC), lysosomal membrane (CC), ion binding (MF), oxidoreductase activity acting on NAD (P)H (MF), and metal ion binding (MF). Full lists of enriched GO annotations after infection with the cp strain after 24 h and 72 h can be found in Tables S6 and S7, respectively.

In turn, the data regarding ncp infection are presented below. Here, 24 h after infection with ncp strains, GO annotations enriched by positively regulated genes (42 terms) with the lowest *p*-values were G-protein-coupled receptor signaling pathway (BP), sensory perception of smell (BP), cell maturation (BP), integral component of membrane (CC), integral component of plasma membrane (CC), plasma membrane (CC), G-protein-coupled receptor activity (MF), olfactory receptor activity (MF), and chemokine activity (MF), while 72 h after infection (27 terms), GO annotations enriched by up regulated genes were L-serine biosynthetic process (BP), reverse cholesterol transport (BP), cholesterol efflux (BP), neuron projection (CC), integral component of plasma membrane (CC), adherens junction (CC), cholesterol transporter activity (MF), hormone activity (MF), and sterol-transporting ATPase activity (MF). Furthermore, 24 h after infection with ncp strains, GO annotations enriched by negatively regulated genes (6 terms) included positive regulation of interferon-gamma production (BP), respiratory chain (CC), extracellular exosome (CC), NADH dehydrogenase (ubiquinone) activity (MF), GTPase activator activity (MF), and growth factor activity (MF), while 72 h after infection (51 terms) GO annotations included proteolysis (BP), immune response (BP), pulmonary artery morphogenesis (BP), extracellular exosome (CC), extracellular space (CC), lysosome (CC), serine-type endopeptidase activity (MF), serine-type carboxypeptidase activity (MF), and calcium-ion binding (MF) (Table 3). Full lists of enriched GO annotations after infection with the ncp strains can be found in Tables S8 and S9.

**Figure 6:**
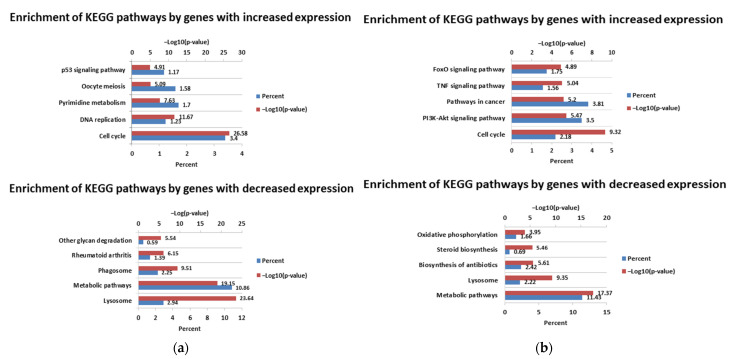
KEGG pathways enriched after infection with cp strain of BVD virus: 24 h p.i. (**a**); 72 h p.i. (**b**).

Another study was concerned the enrichment of KEGG pathways by DEGs. The most significantly enriched pathways 24 h after infection with the cp strain by genes with increased expression were cell cycle, DNA replication, and pyrimidine metabolism, while those significantly enriched by genes with decreased expression included lysosome, metabolic pathways, and phagosome. The most significantly enriched pathways 72 h after infection with the cp strain by genes with increased expression were cell cycle, the PI3K-Akt signaling pathway, and pathways in cancer, while those enriched by genes with reduced expression included metabolic pathways, lysosome, and the biosynthesis of antibiotics. For full lists of enriched pathways, see Tables S10 and S11 (24 h p.i and 72 h p.i., respectively). Figure 6 shows the five most significantly enriched pathways by genes with increased and decreased expression levels after infection with the cp strain at two time intervals.

**Figure 7:**
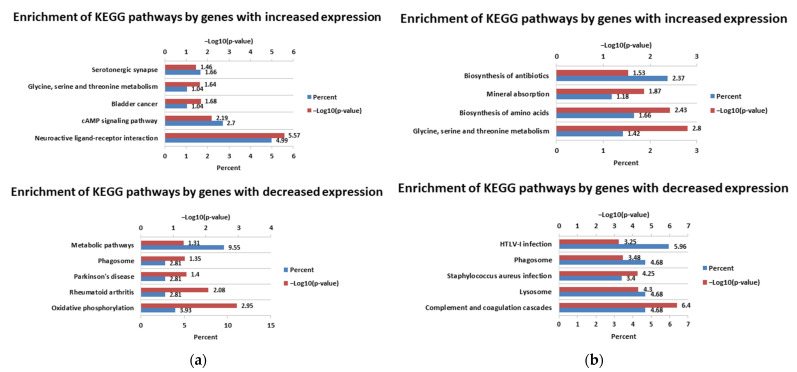
KEGG pathways enriched after infection with ncp strains of BVD virus: 24 h p.i. (**a**); 72 h p.i. (**b**).

The most significantly enriched pathways 24 h after infection with the ncp strains by positively regulated genes were the neuroactive ligand–receptor interaction and cAMP signaling pathway, while those enriched by negatively regulated genes were oxidative phosphorylation and rheumatoid arthritis. The next pathways enriched by genes with increased expression 72 h after infection with ncp strains were glycine, serine and threonine metabolism, and biosynthesis of amino acids. Pathways with the lowest *p*-values enriched by genes with reduced expression 72 h p.i. included complement and coagulation cascades and lysosome. For full lists of enriched pathways, see Tables S12 and S13 (24 h p.i. and 72 h p.i., respectively). Figure 7 shows the five most significantly enriched pathways by genes with increased and decreased expression levels after infections with the ncp strains at two time intervals.

### 3.2. Quantitative Real-Time RT-PCR (RT-qPCR)

**Figure 8:**
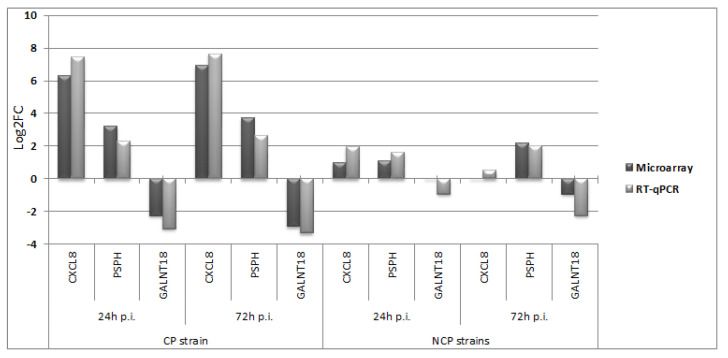
Comparison of gene expression changes in the form of log2FC values obtained with the use of microarrays (dark gray) and RT-qPCR (light gray) for cp and ncp strains of BVDV.

Our studies confirmed the stable expression of B2M in cell cultures uninfected and infected with both biotypes. In the reaction without the enzyme, the Ct value was not obtained, which proved the lack of impurities that could distort the results of the experiment. Figure 8 shows a comparison of the results of gene expression changes generated with microarrays and RT-qPCR. A high correlation was observed between the results obtained by RT-qPCR and microarray methods, with a correlation coefficient of 0.991 (*p* < 0.001).

## 4. Discussion

Microarray and RNA-Seq analyses are the two main techniques used for transcriptome analysis. Analysis of the transcriptome of MDBK cells infected with BVD virus allowed the identification of a number of genes, the expression of which was changed as a result of infection. The experiment used a reference cp NADL strain (subtype 1a) and two Polish field strains belonging to subtypes 1b and 1f. These subtypes (1b and 1f) account for over half of BVD virus strains isolated in Poland [20].

In our study, infection with the cp strain induced changes in the expression levels of a large number of genes. Similarly, a large number of genes showed altered expression as a result of cp infection with the KS86-1cp strain of MDBK cells [17]. Studies by the same authors have shown that BVD virus infection generated highly different gene expression patterns depending on the established cell culture used (MDBK or BFM), indicating significant differences in response to infection in both cell lines [17]. The interstitial cells of Cajal, derived from the duodenum of sheep infected with cp strain BVDV-1b, showed a change in the expression levels of a lower number of genes: 806 genes in total (538 increased and 268 decreased) [21]. It turns out that the BVD virus is able to induce changes in the expression levels of a diverse number of genes, depending on the types of cells in which it multiplies. This has been confirmed by studies performed in different tissues from the same animal [22].

The non-cytopathic strains of BVD virus constitute the vast majority of strains isolated from animals. MDBK cells infected with ncp strain KS86-1ncp showed more gene changes than in our study [17], although peripheral blood mononuclear cells of sheep infected with the ncp strain of BVDV-2 showed changes in the expression of only 449 genes. A total of 97 of those genes showed increased activity and 352 showed decreased activity, although these changes occurred 12 h after infection [23].

The RNA-Seq analysis showed some discrepancies in the number of genes that changed their expression levels at different time intervals from the time of infection. The highest number of genes with altered expression was detected 12 h after infection with the ncp BJ-2016 strain isolated from commercial bovine serum [18]. We also observed differences in the number of genes that changed expression levels with time after infection in our studies. Both the cp and ncp strain infections induced changes in the expression of more genes 24 h after infection than 72 h p.i.; on the other hand, greater changes in gene expression levels (higher absolute FC values) were seen 72 h after infection for both cp and ncp strains.

Transcriptome analyses of infected cells often show significant enrichment of cell cycle pathways, such as in Cajal cells infected with cp strain BVDV [21]. Cell cycle regulation benefits for RNA viruses have been reported, including increased replication, translation, and assembly efficiency of progeny virions [24]. Cell cycle arrest can also delay the apoptosis of infected cells [25]. Our study showed that infection of MDBK cells with a cp strain of BVD virus may cause modifications of the cell cycle, as confirmed by the enrichment of this pathway by 58 genes 24 h after infection (Table S10) and 35 genes after 72 h (Table S11) according to the KEGG database, including changes in expression of cyclins and cyclin-dependent kinases, which are major regulatory proteins of the cell cycle [26]. 

In our study, it was found that the p53 signaling pathway was enriched as a result of infection with the NADL strain (Tables S10 and S11). Enrichment of this pathway was also observed in another study with the ncp strain 12 h after infection [18]. TP53—the p53 gene—was one of the most significant regulators after infection with BVDV-2 [27]. It is a key transcription factor that regulates a number of genes involved in cell cycle arrest, apoptosis, cell aging, DNA repair, metabolism regulation, and autophagy. Furthermore, p53 may contribute to G2 arrest by inducing GADD45A transcription (Tables S2 and S3) (0.75 and 1.46).

The phosphatidylinositol 3’-kinase (PI3K)-Akt signaling pathway was enriched both 24 and 72 h p.i. by the cp strain. This is an intracellular signaling pathway that is important in the regulation of the cell cycle and is activated by many types of stimuli. It regulates basic cellular functions, such as transcription, translation, proliferation, growth, and responses to extracellular signals. Other investigations indicated that PI3K/Akt signaling pathways are induced as a consequence of BoHV-1 infection of MDBK cells, while the activation of PI3K was important for fully efficient replication, especially for the post-entry stage [28].

Many experiments have confirmed that individual HCV proteins are capable of both inhibiting and stimulating various stages of the cycle [29], which may also apply to infections with BVD virus, similarly to HCV, which also belongs to the *Flaviviridae* family.

Infection of MDBK cells with a cytopathic strain leads to cell death, which was directly visible through a light microscope. There were increases in the expression of many transcripts related to cell death, including the intrinsic and extrinsic pathways of apoptosis. ER-stress-induced apoptosis, as well as initiated activation of the cell death receptors, finally activated executive caspases 3 and 7 (1.37 and 0.84 72 h p.i.). Markers of an intrinsic ER-stress-related apoptotic pathway have been activated in the altered mucosa of animals suffering from MD [30], and also in cp-BVDV-infected MDBK cells [17]. Activation of this pathway may, therefore, play a major role in the cell death induced by the cp BVDV strain.

Metabolic processes and pathways are often enriched by DEGs of BVD-infected cells [23]. Many DEGs regulated by BVDV infection have been classified as being involved in metabolic processes in gastrointestinal muscle cells (Cajal cells) infected with the cp strain of BVDV [21]. Some viruses generate an appropriate intracellular microenvironment, promoting their “life cycle” and modulating the cellular metabolic network. 

Our study showed that 72 h after infection with the cp strain, the pathway connected with the metabolism of carbohydrates—glycolysis—was enriched by genes with increased expression (Table S11). On the other hand, the Krebs cycle and phosphorylation oxidation—the stage of final oxidation of organic compounds—were enriched by genes with reduced expression. Oxidative phosphorylation was enriched both 24 and 72 h after infection, while the Krebs cycle was enriched 72 h after infection (Tables S10 and S11). A similar situation occurs in cancer cells directing metabolism to use energy from the faster glycolysis process instead of the more energy-efficient tricarboxylic acid cycle [31]. Although the Krebs cycle is not turned off, it works slower than under normal conditions, resulting in an increase in lactate levels (the so-called Warburg effect). The hepatitis C virus induces glycolysis. Infection of hepatocyte culture cells (Huh-7.5) with this virus reduced the oxidative phosphorylation of host cells while increasing the dependence of metabolism on glycolysis [32]. Although the viral genes necessary for inducing these processes have not been determined, it appears that the viral NS5A protein, by interacting with HK2 (hexokinase 2), increases its activity and may be one of the elements inducing increased glucose uptake and lactic acid production. Cell damage may lead to a reduction in oxidative phosphorylation in the mitochondria, and the level of ATP may be reduced, causing the stimulation of glycolysis.

Additionally, 24 h after infection with the ncp strain, enrichment of the phosphorylation oxidation pathway by genes with reduced expression was identified for genes encoding ATPases, namely ATP6V0A2 (0.6; 0.65) and ATP6V1C2 (0.62–24 h p.i.); components of the respiratory chain, including the NADH dehydrogenase core subunits of the mitochondrial membrane respiratory chain (complex I) 2 (−0.91), 4 L (−0.61), 5 (−0.62), and 6 (−0.61); and the cytochrome c oxidase I subunit (−1.1). Reductions in the expression of genes encoding the core subunits of mitochondrial respiratory chain I (NADH-ubiquinone oxidoreductase) and complex IV (cytochrome c oxidase) were also observed as a result of infection of Huh-7.5 cells with hepatitis C virus [33]. Reduced expression of key genes in the mitochondrial respiratory chains may contribute to the increased flow of metabolites through the glycolysis pathway.

During aerobic glycolysis (also called the Warburg effect), glycerate-3-phosphate produced from glucose is converted to serine by three consecutive enzymatic cascades via phosphoglycerate dehydrogenase (PHGDH), phosphoserine aminotransferase 1 (PSAT-1), and phosphoserine phosphatase (PSPH) [34]. In our study, all genes encoding these proteins increased their expression due to infection with ncp strains PHGDH (0.99; 1.82), PSAT-1 (0.64; 1.28), and PSPH (1.09; 2.17), and the increase in PSPH expression was confirmed by the RT-qPCR test (Figure 6). PHGDH and PSPH also increased their expression due to infection with the cp strain (Tables S2 and S3). There is evidence suggesting that metabolic changes that promote oxygen glycolysis and serine/glycine biosynthesis also occur in HCV-infected cells where PSPH and PSAT-1 expression is significantly increased [35].

In our study, after the infection of MDBK cells with the cp strain, several enriched pathways related to the metabolism of fatty acids (Tables S10 and S11) and the metabolism of lipids, such as steroids or sphingolipids, were identified. Some studies also found that several key genes related to lipid metabolism were regulated during BVDV infection [19]. Other studies have shown that reductions in fatty acid synthesis reduced the production of progeny rotavirus particles [36]. Studies on Dengue infection increased overall fatty acid synthesis in host cells [37]. It is presumed that the modifications of lipid biosynthesis are related to the activation of innate immunity [38].

Studies in BVDV-infected animals have shown the regulation of the lysosome pathway [27]. In our study, reductions in the expression levels of 55 and 32 genes were found 24 and 72 h after infection with the cp strain, respectively, while 11 genes were reduced 72 h p.i. with ncp strains. All of those changes enriched the lysosome pathway according to the KEGG database (Tables S10, S11 and S13). Other studies also indicated enrichment of the lysosome pathway after infection with the NADL (cp) strain [39]. The results of HCV studies indicated that there was no fusion of autophagosomes and lysosomes at an early stage of infection [40]. Additionally, significant dysregulation of lysosome functions was found, confirming the participation of the lysosome in the virus ‘lifecycle’. It is known that the BVDV colocalizes with clathrin, an early endosomal antigen-1, an early endosomal marker, and also with lysosomal membrane protein-2 (LAMP-2), a lysosomal marker [41]. However, further studies are needed to fully understand the role of the modifications of the lysosome pathway during the infection with BVDV.

The innate immunity is the first defense against the agents of infectious diseases. The recognition of PAMPs and the activation of signal transduction pathways are the basis for activating the host’s immune system in response to viral infection. In our study, the signal pathway of the NOD-like receptor (NLR) was enriched by genes positively regulated by NADL strain 24 h and 72 h after infection (Tables S10 and S11). This pathway was also enriched after ncp infection with BVDV-1 [18] and BVDV-2 [23]. Some of the NLRs activate the nuclear factor signaling pathway, NF-κB, which plays an important role in regulating the host immune response, while others act as negative regulators of the NF-κB pathway [42]. Another gene, namely NAIP, whose expression increases (1.07; 1.54), encodes one of the NLRs with antiapoptotic properties [43].

NF-κB is a common transcription factor that controls the expression of a large number of genes involved in important cellular processes. Enrichment of the NF-κB pathway with the cp strain was found 24 h p.i. The activators of the classical pathway are LPS, viruses, and pro-inflammatory cytokines, such as IL-1β and TNFα. The effects of BVDV infection on the NF-κB pathway were found in other studies where Cajal cells were infected with the cp strain [21]. Infection with the cp strain appears to affect the NF-κB signaling pathway. Studies by others revealed that BVD virus NS5A protein inhibited TNF-α- and dsRNA-induced NF-κB activation [12]. We also identified an increase in the expression levels of transcripts related to silencing the NF-κB pathway, such as TNFAIP3 (A20) 24 h after infection (1.91) [44]. The same was also found in MDBK cells infected with BVDV-1, 2, and 4 at 24 h p.i. [45]. There was also an increase in the expression of genes encoding NF-κB inhibitors, such as NFKBIA (2.23; 2.44), by cp (Tables S2 and S3) and ncp strains 24 h p.i. (0.67) (Table S4). The inhibition of the NF-κB pathway may be responsible for the immunosuppression that occurs during BVD virus infection.

Inflammation is one of the host’s most important defense mechanisms against microorganisms. Host cells exposed to infectious agents produce cytokines, such as TNF-α, IL-1, and IL-6, stimulating the development of inflammation [23]. The mRNA levels of BVDV-infected cells indicated the induction of an acute inflammatory response at an early stage of infection [23,27]. Other studies indicate that both cp and ncp strains can suppress the pro-inflammatory cytokines TNF-α, IL-1β, and IL-6 [46]. In our studies, some mRNAs encoding these proteins increased their expression levels only after being infected with the cp strain. TNF-α is an essential mediator of inflammation that also facilitates the transition from innate to adaptive immunity. Enrichment of the TNF pathway was found after infection with the cp strain. TNFR1 signaling induces the activation of many genes, mostly controlled by two distinct pathways, the NF-kappa B pathway and the MAPK cascade, or apoptosis and necroptosis [47].

In our study, the cp strain induced the expression of the cytokines G-CSF (CSF3; 4.33; 1.38) and CSF2 (GM-CSF; 3.22; 3.82) and the chemokines CXCL1 (7.2; 7.59), CXCL8 (6.31; 6.93), CXCL3 (4.48; 4.56), and CXCL5 (3.77; 2.97). GM-CSF expression was also increased following infection of peripheral blood mononuclear cells by BVDV [23]. Genes encoding CXCL1 (GRO1) and CXCL8 (IL-8) showed the highest changes in expression in cells infected with the NADL (cp) strain, which in the case of CXCL8 was confirmed by RT-qPCR, while the chemokine signaling pathway was enriched by genes with increased expression 72 h after infection (Table S11). In this study, the expression of CXCL8 changed over 79 and over 122 times, at 24 and 72 h p.i., respectively. Additionally, Yamane et al. [17] described a more than 50-fold increase in IL-8 expression after cp BVDV infection in MDBK cells. In another study using Cajal cells, the expression of CXCL8 was increased by the cp TC strain by more than 1000-fold, confirming that infection with the cp strain induced a significant response at the transcriptome level [21]. The increase in many signaling pathways related to the biosynthesis and actions of cytokines and chemokines, such as the cytokine−cytokine interaction pathway, TNF signaling pathway, and chemokine signaling pathway, was observed after the infection of Cajal cells [21] and blood-nucleated cells [23]. An increase in the expression of chemokines and cytokines was also observed in BFM and MDBK cells infected with cp strain [17]. ELR-type chemokines activated by cp strain infection included CXCL1, CXCL3, CXCL5, CXCL8, and CXCL15. These are expressed in infected tissues, exhibit angiogenic and chemotactic properties for neutrophils, and are bound by CXCR2 receptors. Their expression is stimulated by various signals, including hypoxia; oxidative stress; the pro-inflammatory cytokines TNF-α, IL-1, IFN-γ, and IL-6; and thrombin [48].

Transcriptome studies have indicated increased transcription of serum amyloid A3 SAA3 [23]. Similarly, Gånheim et al. found upregulation of SAA and other acute phase proteins in calves experimentally infected with BVDV [49]. In our studies, after infection of MDBK cultures with ncp strains of the BVD virus, the SAA3 gene showed the greatest change in expression 24 h after infection (1.95), while after 72 h p.i., its expression showed no significant changes. We observed a similar pattern after inoculation with the cp strain, which increased the expression of SAA2 (3.77; 1.03) and SAA3 (3.39; 1.2).

Our study did not show any noticeable antiviral innate immune response in MDBK cells inoculated with the ncp strains. Interferon-stimulated genes are important components of the host’s innate immune system, which play a key role in the defense mechanism against viral infection [50]. According to other authors, individual ISGs were differently expressed during BVDV infection; some decreased their expression [18] and others showed weak ISG induction in both BFM and MDBK cells [17] or a lack of interferon expression and ISG influence by BVDV-2 infection [23]. The inhibition of IFN synthesis plays an important role in the virus escaping from the body’s innate immunity and in the successful infection of the host cells. “Innate tolerance” to BVDV may not be limited only to the system associated with IFN synthesis, but also to other pathways and components of innate immunity. Non-cytopathic BVDV strains, such as strain 890, do not induce or even inhibit the synthesis of pro-inflammatory cytokines and costimulatory molecules [22].

In our study, some components enriched the complement pathway 72 h p.i. with the ncp and cp strains (Tables S11 and S13). Component C3 was one of the main genes with reduced expression 72 h after infection with the ncp strain (Table S5). In the studies by Li et al., decreased mRNA levels were found for genes encoding components of the complement system, such as C1q, C1r, C1s, and CFD [23]. Furthermore, changes in the expression levels of genes, such as F3, C1R, KNG1, CLU, C3, FB, SERPINA5, SERPINE1, C1S, F2RL2, and C2, which belong to the complement and coagulation signaling cascades, were detected by Liu et al. [18], emphasizing that the complement system might play a crucial role during BVDV infection. Studies in BVD-virus-infected animals confirmed the significant enrichment of the complement and coagulation cascade compared to control animals [27]. Previous studies showed that BVD virus E2 glycoprotein, produced in both soluble and transmembrane form in stable CHO-K1 (hamster ovary) cell lines, was able to reduce cell lysis mediated by the complement system [51]. It is also known that complement activation is inhibited by the HCV NS3/NS4A protease [52]. C3 expression also reduces the hepatitis C core protein by silencing expression of the transcription factor FXR. C3 expression is also strongly decreased by NS5A protein by reducing the expression of IL-1β, which promotes C3 expression [53]. 

Neuroactive ligand–receptor interaction was enriched 24 h after infection with ncp strains. This involves a collection of signaling molecules, such as hormones, neurotransmitters, and their corresponding receptors, as well as several classes of G-protein-coupled receptors, e.g., dopamine, 5-hydroxytryptamine (5-HT) and histamine receptors, and γ-aminobutyric acid (GABA).

Similarly, the cAMP signaling pathway was enriched 24 h p.i. with ncp strains. Furthermore, cAMP is one of the most common and universal messenger activators after the ligation of G-protein-coupled receptors by ligands, including hormones, neurotransmitters, and other signaling molecules; cAMP regulates pivotal physiologic processes, including metabolism, secretion, calcium homeostasis, muscle contraction, cell fate, and gene transcription [54].

It is worth mentioning the increased expression of metallothioneins (MT) by the ncp and cp strains (Tables S2–S5). MT are low molecular weight cysteine-rich proteins that physiologically bind zinc and copper in cells. Reductions in blood zinc concentration have been observed in the course of various infectious diseases [55]. The research results suggest that, under inflammatory conditions, metallothioneins in the extracellular environment may aid in leukocyte translocation to the site of inflammation [56]. The A/PR8 strain of the influenza virus has been shown to be able to potentially activate MT1 and MT2 expression in the lungs and liver, possibly to limit the oxidative damage caused by inflammation [57]. Subsequent studies showed that zinc-secreting compounds inhibit the activity of M2-1 protein, an essential cofactor that prevents premature termination of transcription of the RNA-dependent viral RNA polymerase complex by increasing the enzyme activity [58]. ZnSO4 has been shown to reduce HCV replication [59]. The antiviral effect of ZnSO4 was abolished when metallothionein genes were blocked by siRNA. Supplementation of animals with trace elements may affect the functioning of the immune system and reduce the incidence of viral respiratory diseases [60].

## 5. Conclusions

In the present study, we characterized the transcriptome profiles of MDBK cells infected with BVDV using microarrays. We showed that changes in the expression levels of many genes that can play an important role in the pathogenesis of BVDV. Our study suggests that many DEGs were involved in altering the host’s cell cycle, metabolic network, signaling pathways, and innate immune response, with large differences depending on the viral biotype. The inhibition of the expression of DEGs related to the innate immune response, for example, related to complement cascade, may partly explain the increased susceptibility of cattle to secondary infections. The data from this study can help us to better understand the mechanisms underlying BVDV–host interactions on the transcriptome level, although insights into fully recognized functional changes in MDBK cells infected with BVDV require more research.

## Figures and Tables

**Table 1:** Sequences of primers used in the RT-qPCR to confirm the results of the microarray analysis. * Gene used as reference.

Gene	Primer	Sequence	Reference
B2M *	B2M-F	TCGTGGCCTTGGTCCTTCT	Elgendy et al., 2017 [16]
	B2M-R	AATCTTTGGAGGACGCTGGAT	
IL-8	IL-8-F	TGAAGCTGCAGTTCTGTC	Yamane et al., 2009 [17]
	IL-8-R	ATTTGGGGTGGAAAGGTG	
PSPH	PSPH-F	TCAAGGCTGCCCTCACACA	Liu et al., 2019 [18]
	PSPH-R	AGGAGCCTCTGCACCTGTTC	
GALNT18	GALNT18-F	GTGCCGCAACCTCTCGTT	
	GALNT18-R	CACGGAGAGTGCTTCATTGACA	

**Table 2:** GO terms enriched 24 and 72 h p.i. with cp strain of BVD virus.

	Enrichment of GO Terms by Genes with Changed Expression 24 h p.i.			Enrichment of GO Terms by Genes with Changed Expression 72 h p.i.		
	Enrichment of GO terms by genes with increased expression					
Category	Term	Count	*p*-value	Term	Count	*p*-value
biological process	cell division	60	2.17 × 10^−^^22^	cell division	36	1.64 × 10^−7^
	mitotic nuclear division	38	9.40 × 10^−^^13^	regulation of cell proliferation	35	2.58 × 10^−7^
	cell cycle	29	3.85 × 10^−^^8^	apoptotic process	43	4.11 × 10^−7^
cellular component	cytoplasm	413	2.25 × 10^−^^15^	cytoplasm	369	1.23 × 10^−9^
	nucleus	385	9.02 × 10^−^^15^	nucleus	332	2.19 × 10^−7^
	spindle pole	31	1.48 × 10^−^^12^	spindle pole	22	1.73 × 10^−6^
molecular function	ATP binding	172	2.85 × 10^−^^8^	microtubule binding	20	9.85 × 10^−6^
	DNA binding	101	1.75 × 10^−^^7^	ATP binding	146	1.86 × 10^−4^
	histone binding	16	5.94 × 10^−^^6^	protein kinase binding	19	1.97 × 10^−4^
	Enrichment of GO terms by genes with decreased expression					
biological process	sphingolipid biosynthetic process	9	8.48 × 10^−6^	metabolic process	20	8.70 × 10^−5^
	antigen processing and presentation of peptide or polysaccharide antigen via MHC class II	10	2.19 × 10^−5^	ATP synthesis coupled electron transport	5	5.12 × 10^−4^
	protein glycosylation	21	4.46 × 10^−5^	response to oxidative stress	14	8.06 × 10^−4^
cellular component	lysosomal membrane	62	2.11 × 10^−18^	extracellular exosome	247	1.29 × 10^−10^
	extracellular exosome	336	5.33 × 10^−18^	lysosome	33	2.92 × 10^−8^
	lysosome	44	1.47 × 10^−11^	lysosomal membrane	36	3.92 × 10^−7^
molecular function	monooxygenase activity	12	1.38 × 10^−4^	ion binding	287	3.85 × 10^−4^
	heme binding	26	2.01 × 10^−4^	oxidoreductase activity, acting on NAD(P)H	16	7.17 × 10^−4^
	serine-type carboxypeptidase activity	7	2.57 × 10^−4^	metal ion binding	272	7.26 × 10^−4^

**Table 3:** GO terms enriched 24 and 72 h p.i. with ncp strains of BVD virus.

	Enrichment of GO Terms by Genes with Changed Expression 24 h p.i.			Enrichment of GO Terms by Genes with Changed Expression 72 h p.i.		
	Enrichment of GO terms by genes with increased expression					
Category	Term	Count	*p*-value	Term	Count	*p*-value
biological process	G-protein coupled receptor signaling pathway	40	2.43 × 10^−5^	L-serine biosynthetic process	3	0.001510151
	sensory perception of smell	16	2.13 × 10^−4^	reverse cholesterol transport	4	0.001657372
	cell maturation	6	0.001282379	cholesterol efflux	5	0.001914924
cellular component	integral component of membrane	168	1.20 × 10^−10^	neuron projection	10	0.002921923
	integral component of plasma membrane	50	4.05 × 10^−8^	integral component of plasma membrane	29	0.021268826
	plasma membrane	93	4.63 × 10^−6^	adherens junction	4	0.032851986
molecular function	G-protein coupled receptor activity	47	2.61 × 10^−5^	cholesterol transporter activity	5	1.07 × 10^−04^
	olfactory receptor activity	35	0.002688453	hormone activity	5	0.038843617
	chemokine activity	5	0.014352821	sterol-transporting ATPase activity	2	0.044400249
	Enrichment of GO terms by genes with decreased expression					
biological process	positive regulation of interferon-gamma production	4	0.005107499	proteolysis	10	0.001012604
	-	10	2.19 × 10^−5^	immune response	11	0.001159766
	-	21	4.46 × 10^−5^	pulmonary artery morphogenesis	3	0.002023761
cellular component	respiratory chain	4	3.32 × 10^−4^	extracellular exosome	66	5.00 × 10^−10^
	extracellular exosome	29	0.042136161	extracellular space	34	1.01 × 10^−6^
	-	44	1.47 × 10^−11^	lysosome	10	1.58 × 10^−4^
molecular function	NADH dehydrogenase (ubiquinone) activity	5	2.09 × 10^−4^	serine-type endopeptidase activity	11	8.04 × 10^−5^
	GTPase activator activity	6	0.024894456	serine-type carboxypeptidase activity	4	4.69 × 10^−4^
	growth factor activity	4	0.027131177	calcium ion binding	20	7.32 × 10^−4^

## Data Availability

Source of datasets used in this study are available at GEO with the accession numbers GSE166288 and GSE166528.

## Supplementary Materials

The following are available online at https://www.mdpi.com/article/10.3390/v14061276/s1: Figure S1: PCA—cp strain; Table S1: Differentially expressed entities between infection with subtype 1f and 1b; Table S2: Differentially expressed entities 24 h p.i. with cp; Table S3: Differentially expressed entities 72 h p.i. with cp; Table S4: Differentially expressed entities 24 h p.i. with ncp; Table S5: Differentially expressed entities 72 h p.i. with ncp; Table S6: GO enrichment results of the DEGs 24 h p.i. with cp strain; Table S7: GO enrichment results of the DEGs 72 h p.i. with cp strain; Table S8: GO enrichment results of the DEGs 24 h p.i. with ncp strains; Table S9: GO enrichment results of the DEGs 72 h p.i. with ncp strains; Table S10: KEGG pathway enrichment result of the DEGs 24 h p.i. with cp strain; Table S11: KEGG pathway enrichment result of the DEGs 72 h p.i. with cp strain; Table S12: KEGG pathway enrichment result of the DEGs 24 h p.i. with ncp strains; Table S13: KEGG pathway enrichment result of the DEGs 72 h p.i. with ncp strains.

## Abbreviations

ATCC: American Type Culture Collection; BFM: bovine fetal muscle; BP: biological process; BVDV: bovine viral diarrhea virus; B2M: beta-2-microglobulin; cAMP: 3’,5’-cyclic adenosine monophosphate; CC: cellular component; cp: cytopathic; Ct: cycle threshold; Cy: cyanine; DEGs: differentially expressed genes; ER: endoplasmatic reticulum; FC: fold change; FDR: false discovery rate; GALNT18: polypeptide N-acetylgalactosaminyltransferase 18; GO: Gene Ontology; HCV: hepatitis C virus; IFN: interferon; IL: interleukin; ISGs: interferon-stimulated genes; KEGG: Kyoto Encyclopedia of Genes and Genomes; MAPK: mitogen-activated protein kinases; MDBK: Madin–Darby bovine kidney; MF: molecular function; MOI: multiplicity of infection; MD: mucosal disease; MT: matalothionein; ncp: non-cytophatic; NF-kB: nuclear factor kappa B; NLR: NOD-like receptor; PAMPs: pathogen-associated molecular patterns; PCA: principal component analysis; PBMCs: peripheral blood mononuclear cells; PDPH: phosphoserine phosphatase; PHGDH: phosphoglycerate dehydrogenase; PI3K: phosphoinositide 3-kinase; p.i.: post-infection; RIN: RNA integrity number; RT-qPCR: reverse-transcription quantitative polymerase chain reaction; SAA: serum amyloid A; TLRs: Toll-like receptors; TNF: tumor necrosis factor.
