# Supplementary material for: Transcriptomic Analysis of MDBK Cells Infected with Cytopathic and Non-Cytopathic Strains of Bovine Viral Diarrhea Virus (BVDV)
Source: Viruses. 2022 Jun 11;14(6):1276. doi: 10.3390/v14061276 (PMC9228727; doi:10.3390/v14061276)
Supplement: Supplementary file 1 [file viruses-14-01276-s001.zip › Figure S1_PCA - cp strain.pdf]

a)

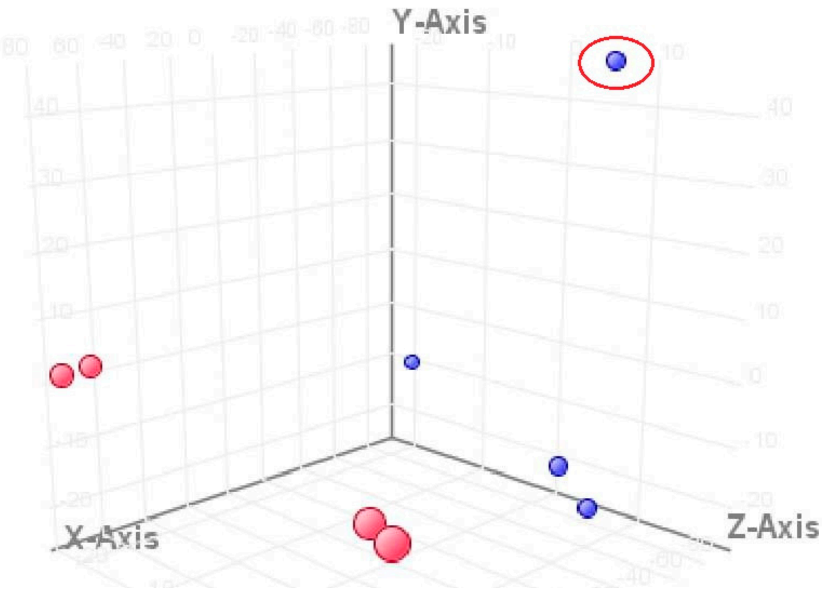

b)

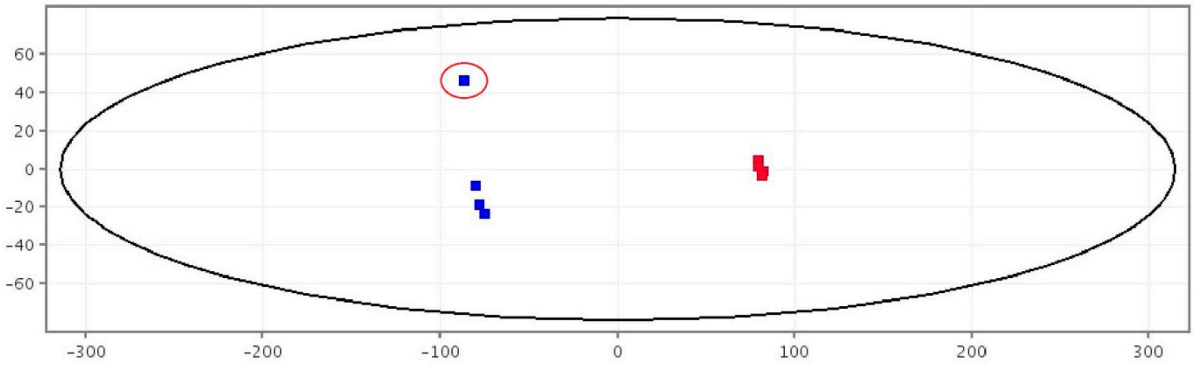

Analysis of the main sources of variation by PCA method between the microarrays made for cells infected with the cp strain using three main components **(a)** and two main components **(b)**. The red dots represent the gene expression profiles of MDBK cells 24h p.i. with cp strain, blue dots 72h p.i. A red oval marks the sample that was unsealed during hybridization.
